# Evaluation of serologic assays for detection and quantification of influenza antibodies in milk samples from H5N1 highly pathogenic avian influenza virus infected dairy cows

**DOI:** 10.3389/fvets.2026.1902594

**Published:** 2026-07-28

**Authors:** Sherif Ibrahim, Youngsub Lee, Erin Posey, Mia K. Torchetti, Tim Olivier, Erica Spackman, David L. Suarez, Chang-Won Lee

**Affiliations:** 1 Oak Ridge Institute for Science and Education, Oak Ridge Associated Universities, Oak Ridge, TN, United States; 2 Exotic and Emerging Avian Viral Diseases Unit, Southeast Poultry Research Laboratory, U.S. National Poultry Research Center, Agricultural Research Service, U.S. Department of Agriculture, Athens, GA, United States; 3 National Veterinary Services Laboratories, Animal and Plant Health Inspection Service, U.S. Department of Agriculture, Ames, IA, United States

**Keywords:** antibody response, bovine milk sample, enzyme-linked lectin assay, highly pathogenic avian influenza, neuraminidase inhibition antibody

## Abstract

Since the confirmation of H5N1 HPAI virus in US dairy cattle on March 25, 2024, the bovine HPAI outbreak has continued with more than 1,000 confirmed herds in 19 states reported as of April 2, 2026. To ensure ongoing monitoring of HPAI spread, additional serologic assays have been evaluated to complement existing commercial ELISAs and molecular tools that underpin current epidemiological approaches. We previously established an N1 antibody-specific neuraminidase inhibition (NI)-enzyme linked lectin assay (ELLA) for DIVA (differentiation of infected from vaccinated animals) surveillance to detect H5N1 infection in vaccinated poultry with inactivated heterologous NA subtype (H5N9) vaccine. In this study, we applied NI-ELLA for the detection and quantification of N1 antibody in individual (*n* = 110) and bulk tank (*n* = 104) bovine milk samples. We demonstrated that both untreated samples and infranatant collected by centrifugation followed by heat inactivation can be used for different serologic assays depending on sample quality. There was a strong correlation in antibody detection between NI-ELLA and commercial ELISAs. Endpoint titers determined by NI-ELLA also correlated well with virus neutralization titers, which are used to evaluate protective antibody levels in milk samples. Detection and quantification of N1 antibodies in bulk tank milk samples further showed higher detection rates and stronger antibody titers among virus isolation-negative samples (regardless of the presence or absence of viral RNA detected by PCR) compared to virus isolation-positive samples. Higher levels of neutralizing antibody likely contributed to the lack of infectious virus detection depending on the stage of viral infection when milk was collected. Future studies are warranted to describe the duration and dynamics of antibody responses in milk samples over extended periods across individual cows, and NI-ELLA will serve as a valuable quantitative tool for evaluating antibody responses in those studies.

## 1 Introduction

Clade 2.3.4.4b H5N1 highly pathogenic avian influenza (HPAI) viruses of the goose/Guangdong/1996-like lineage have spread globally since 2020 (1) and, unexpectedly, established sustained transmission in United States (US) dairy cattle (2). H5N1 HPAI virus infection in dairy cattle, although primarily localized to the mammary gland, can cause severe disease with some mortality, greatly reduce both the quantity and quality of milk production, and lead to earlier culling from the dairy farm (3). Since the first detection of H5N1 virus in commercial dairy herds in Texas in March 2024, the virus has also been detected in peridomestic birds, cats, mice, and several other mammals, including humans (4, 5). Effective surveillance and containment measures are essential to prevent and control outbreaks, reduce potential zoonotic transmission, and maintain sustainable dairy operations.

Earlier reports from the late 1990s and 2000s suggested a possible association between influenza A virus (IAV) antibody detection and decreased milk production in dairy cows (6–8). However, the current H5N1 HPAI virus infections in dairy cattle differ substantially due to both the severity of disease and the high viral loads associated with infection (9–11). Experimental challenge studies and analyses of IAV receptor distribution and virus binding pattern show acute, efficient replication of H5N1 virus in the mammary gland correlating with wide expression of α2,3-linked sialic acid receptors and the presence of viral antigen in mammary alveolar epithelial and intraluminal cells (9, 12, 13).

Milk is a valuable diagnostic analyte that can identify active infection or prior pathogen exposure (14). Detection of HPAI virus and/or specific antibodies in milk is used for monitoring HPAI virus infection at on-farm, county, state, and national-levels and for informing decisions related to HPAI control and management. Bulk tank milk samples are a cost-effective aggregate sample used to assess disease status for whole herds on dairy farms (14, 15). In the US, a voluntary national dairy herd monitoring program has tested weekly bulk tank milk samples for HPAI virus since 2024 (16).

Reliable diagnostic and surveillance tools are crucial for effective HPAI control. Due to its high sensitivity and specificity for detecting active infection, real-time reverse-transcription PCR (rRT-PCR) has been used by the National Animal Health Laboratory Network (NAHLN) for detection of H5N1 in US livestock. Although rRT-PCR is effective for identifying active infections, an antibody-based approach provides complementary value for surveillance. Previous studies have demonstrated the effectiveness of nucleoprotein (NP)-based competitive enzyme-linked immunosorbent assays (ELISAs) and H5-based fluorescent microsphere immunoassay (FMIA) for detecting H5N1 antibodies in dairy cattle (17, 18). In experimental pathogenesis and vaccine studies, hemagglutination inhibition (HI) and virus neutralization (VN) tests are traditionally performed using serum samples, with VN tests being used for milk samples. These assays measure antibody levels and correlate with protective immunity from infection or vaccination (12, 19). Enzyme-linked lectin assay (ELLA) measures neuraminidase activity and can be adapted to evaluate neuraminidase inhibition (NI) antibody levels (20–22). We recently developed an N1-specific NI-ELLA to detect H5N1 virus infection in poultry and demonstrated its potential for DIVA (differentiation of infected from vaccinated animals) surveillance (21, 23–25).

In this study, we evaluated NI-ELLA for detection and quantification of N1 antibody in individual cow milk samples from H5N1 virus infected herds and in aggregate bulk tank milk samples and compared its performance with other serologic tests. Our findings show that NI-ELLA is an effective tool for H5N1 HPAI infection monitoring in dairy cattle and for evaluating antibody responses in milk samples relative to infection stage and protective immunity.

## 2 Materials and methods

### 2.1 Milk samples

Milk samples from individual cows (independent samples from different cows) were collected in 2024 from a dairy farm with suspected cases of H5N1 2.3.4.4b based on clinical signs. Samples were initially tested by rRT-PCR following a NAHLN-approved method (26) and by a commercial ELISA (IDEXX Laboratories Inc., Westbrook, ME) using a validated, modified manufacturer protocol at the National Veterinary Services Laboratories (NVSL, Ames, Iowa). Virus isolation was conducted by propagating the samples in specific pathogen free embryonating chicken eggs and virus isolation negative samples (*n* = 110), confirmed by hemagglutination (HA) negative testing of allantoic fluid, were selected and shipped to the Southeast Poultry Research Laboratory, U.S. National Poultry Research Center, USDA-Agricultural Research Service (Athens, Georgia) for further serologic analysis using multiple assays in biosafety level 2 (BSL-2) condition. Milk samples (*n* = 40) collected from farms not known to be affected by HPAI and confirmed H5 antibody negative were used as negative controls to assess nonspecific reactivity in serologic assays.

Bulk tank milk samples were previously collected for a different study over a two-week period from four states (27). Samples were taken from farms known to be affected by HPAI as well as farms not known to be affected. Selected samples (*n* = 104), consisting of PCR positive and virus isolation positive samples (*n* = 38), PCR positive and virus isolation negative samples (*n* = 43), and PCR negative samples (*n* = 23), all stored at −80 °C, were used in this study.

### 2.2 Sample preparation

Untreated samples, infranatant samples (the clear milk fraction below the fat layer collected after centrifugation), and heat-treated infranatant samples were tested depending on the assays. Milk samples were centrifuged at 13,000 × g for 15 min at 4 °C to remove the upper fat layer. The infranatant was collected and heat-treated at 56 °C for one hour.

### 2.3 Enzyme-linked immunosorbent assay

Three commercial ELISAs, IDEXX AI MultiS-Screen (IDEXX-NP; IDEXX Laboratories Inc., Westbrook, ME), ID Screen Influenza A Antibody Competition Multi-species (IDvet-NP; Innovative Diagnostics, Grabels, France), and ID Screen Influenza H5 Antibody Competition 3.0 Multi-species (IDvet-H5; Innovative Diagnostics) were used and results were interpreted according to the manufacturers’ instructions. All ELISAs used in this study are competitive or blocking ELISAs and antibody presence is determined by the sample to negative (S/N) ratio for each specimen. For easier comparison of results derived from different ELISAs and other serologic tests, percent inhibition (PI) values were calculated by subtracting the competition percentage [S/N% = (sample OD / negative control OD) × 100] from 100%. Both IDEXX-NP and IDvet-NP ELISAs detect antibodies against influenza A virus nucleoprotein (NP), whereas the IDvet-H5 ELISA detects antibodies against the H5 subtype hemagglutinin.

### 2.4 Enzyme-linked lectin assay (ELLA) for measurement of neuraminidase inhibition (NI) antibody (NI-ELLA)

The N1 specific NI-ELLA was performed using the inactivated H7N1 virus (A/Flycatcher/CA/14875-1/94) as previously established for chicken and cattle sera (21, 28). The detailed antigen information, including the N1 sequence and its reactivity to various N1 and other NA subtypes, as well as the assay parameters, and performance metrics, were previously reported (21). Briefly, ELLAs were conducted using MaxiSorp, clear flat-bottom 96-well plates (Thermo Fisher Scientific, Waltham, MA) coated with 25 μg/mL fetuin (100 μL/well; Sigma-Aldrich, Inc.). Milk samples were tested either at a 1:20 and 1:40 dilution or in serial two-fold dilutions (starting at 1:20) for titration in a dilution plate and mixed with an equal volume of antigen (98.5% effective concentration, EC98.5), which had been previously established under the same protocol (28). H5N1 positive and negative control bovine serum samples, provided by the NVSL, were included to validate the test. Fetuin-coated plates were washed three times with Dulbecco’s phosphate buffered saline (DPBS) containing 0.05% Tween 20 (washing buffer), and the milk-virus mixture was added and incubated at 37 °C in a humidified chamber overnight for 19–20 h. Plates were washed six times with washing buffer. Then, 100 μL of peanut agglutinin horseradish peroxidase (PNA-HRP) conjugate (Sigma-Aldrich) in DPBS with CaCl_2_ and MgCl_2_ containing 1% bovine serum albumin (conjugate buffer) was added to each well. After a 2 h incubation in the dark at room temperature (approximately 23 °C), plates were washed three times with washing buffer, developed for 10 min with TMB ELISA substrate (Thermo Fisher Scientific), and the reaction was stopped with ELISA stop solution (Thermo Fisher Scientific). Plates were read at an optical density of 450 nm using a SpectraMax Multi-Mode Microplate Reader (Molecular Devices, Sunnyvale, CA) or a Synergy HTX multimode reader (BioTek, Winooski, VT). Percent NI activity for each milk sample was determined by subtracting percent NA activity from 100%.

### 2.5 Virus neutralization (VN) test

Two-fold serial dilutions of each sample (1:10–1:1,280) were incubated with 100 50% tissue culture infectious doses (TCID_50_)/50 μL of A/dairy cattle/Texas/24-008749-002/2024 (TX/24, H5N1, genotype B3.13) virus in 96-well dilution plates. H5N1 positive and negative control bovine serum samples were included in the test. After one hour of incubation, 100 μL of the virus-sample mixture was transferred to 96-well culture plates seeded with Madin-Darby canine kidney (MDCK) cells. After 3 days of incubation at 37 °C, cells were examined for cytopathic effects (CPE) as evidence of viral replication. Milk VN titers were expressed as the reciprocal of the highest dilution that completely prevented CPE.

### 2.6 Statistical analysis

Test results were analyzed using Prism 10 (GraphPad Software LLC, Boston, MA), and *p*-values < 0.05 were considered statistically significant. Differences in antibody responses among treatment, sample, or serologic test groups were evaluated using either Student’s *t*-test or one-way ANOVA, depending on the number of groups. Pearson correlation (two-tailed) was used to compare antibody titers obtained from different serologic assays.

## 3 Results

### 3.1 Effect of sample processing/treatment on non-specific reactivity in NI-ELLA

**Figure 1:**
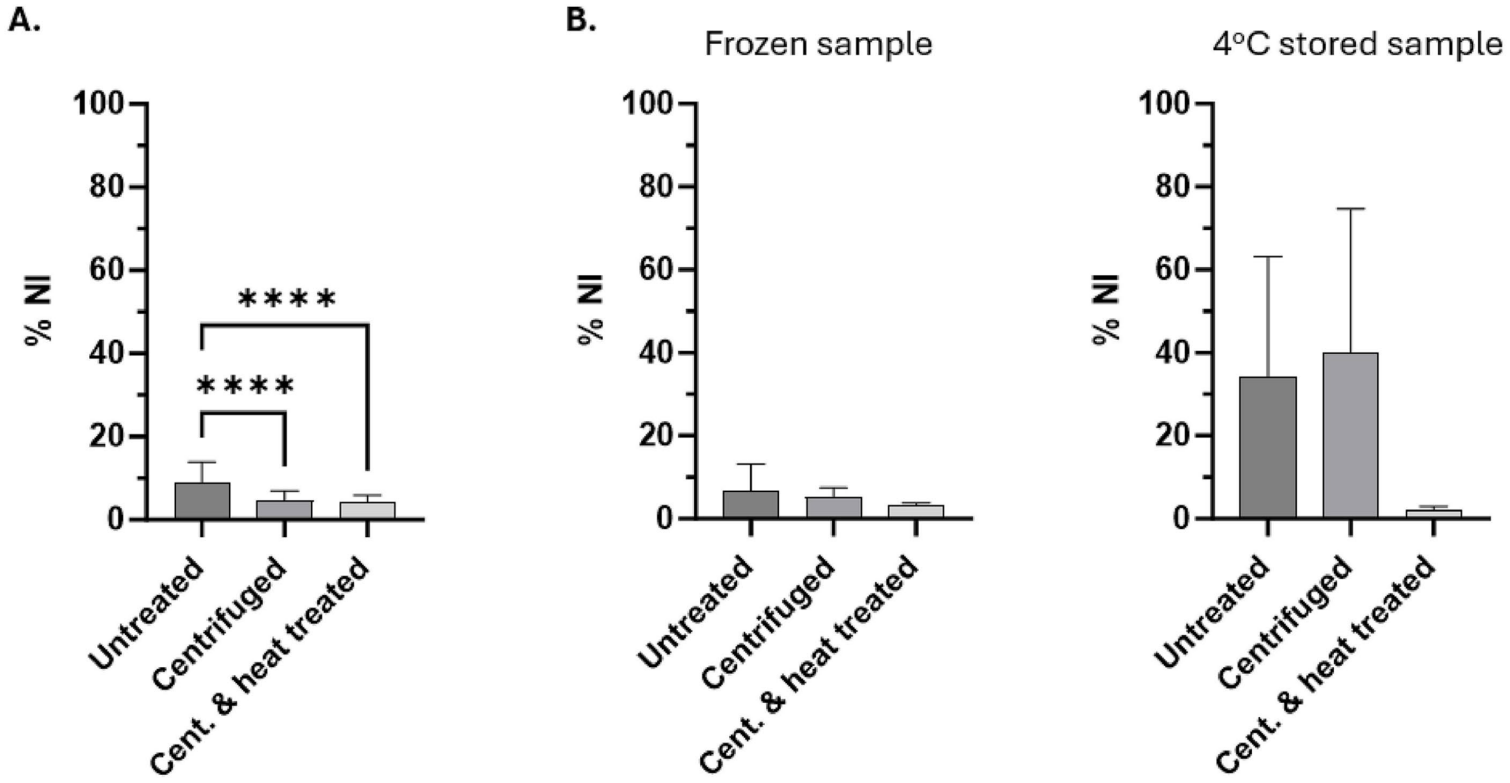
Effect of sample treatment on nonspecific reactivity of the neuraminidase inhibition - enzyme linked lectin assay (NI-ELLA) evaluated using negative control milk samples collected from dairy herds not infected with highly pathogenic avian influenza (HPAI) virus (**A**, *n* = 40) and influenza A antibody-negative samples collected from HPAI virus infected herds (**B**, *n* = 4). In panel B, duplicate samples stored at −80 °C or 4 °C for more than a month were compared. NI-ELLA was performed using samples that were untreated, centrifuged at 13000 × g (infranatant collected and used), or centrifuged and heat-treated (infranatant heat-inactivated at 56 °C for one hour). Repeated-measures ANOVA was used to compare the percent NI activities of tested samples across different treatments, followed by Tukey’s post-hoc pairwise comparisons for significant ANOVA (Prism 10, GraphPad Software LLC). ****p < 0.0001.

To assess nonspecific reactivity (background noise) of milk samples in NI-ELLA, we compared three different preparations of 40 negative-control milk samples collected from dairy herds not infected with HPAI virus (Figure 1A). Newly thawed samples without any further processing or treatment (untreated sample) showed less than 10% mean NI reactivity at a 1:20 dilution, which is generally considered acceptable depending on study purpose (20, 21). Infranatant collected after high-speed centrifugation (centrifuged sample) significantly reduced background nonspecific reactivity (*p* < 0.0001). Additional heat treatment of the infranatant (centrifuged and heat-treated samples) showed a similar level of low background reactivity as observed with just the centrifuged samples.

Four HPAI virus-negative (IDEXX NP-ELISA negative) samples collected from HPAI infected herds, stored either frozen or at 4 °C for over a month, were also compared (Figure 1B). Interestingly, frozen samples exhibited patterns of nonspecific reactivity similar to negative control samples (Figure 1A), whereas samples stored at 4 °C displayed higher nonspecific reactivity (approximately 40%) in both untreated and centrifuged preparations. Although statistical analyses did not show significant difference due to the small number of samples, heat treatment of infranatant reduced nonspecific reactivity to below 5% in all cases. Because quality of milk samples can vary widely depending on the source, storage, number of freeze–thaw cycles, etc., centrifugation alone may be insufficient to control nonspecific reactivity; therefore, untreated versus centrifuged/heat-treated milk samples were compared further in the following experiment.

### 3.2 Detection of N1 specific NI antibody in milk samples

**Figure 2:**
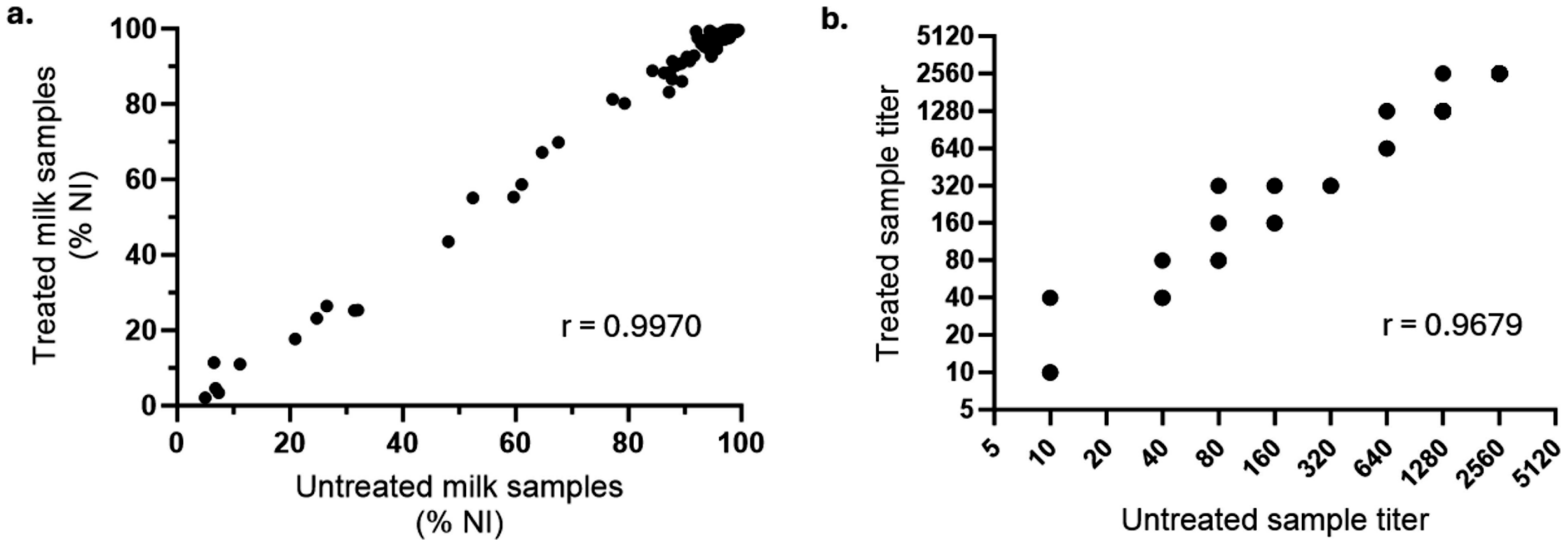
Detection, endpoint titration, and comparison of N1-specific antibody in milk samples and comparison between sample treatments. **(A)**. Percent neuraminidase inhibition (NI) measured using 1:20 dilutions of matching untreated and treated (heat treated infranatant) milk samples (*n* = 110). **(B)**. Fifty percent endpoint titers (last dilution showing ≥50% NI activity) of selected treated and untreated milk samples (*n* = 41). Any titer below 20 was assigned a value of 10. Pearson correlation coefficients (*r*) (two-tailed test) are shown.

Although centrifugation and heat treatment reduce nonspecific reactivity of NI-ELLA, additional treatment of samples may also affect assay sensitivity. To assess effect of sample treatment on NI-ELLA sensitivity, 110 samples (untreated or centrifuged/heat-treated) collected from HPAI infected dairy herds were tested at a 1:20 dilution. As shown in Figure 2A, percent NI activity between matching samples of different treatments was nearly identical with a Pearson correlation coefficient (*r*) of 0.9970. Fifty percent endpoint titration of selected samples (*n* = 41) also resulted in similar titers with no significant difference between the treatments and a high correlation between the titers (*r* = 0.9679; Figure 2B). Thus, additional processing (centrifugation and heat-treatment) of samples reduces nonspecific reactivity without compromising N1-specific NI antibody detection.

### 3.3 Comparison of commercial ELISAs and NI-ELLA using selected milk samples

**Figure 3:**
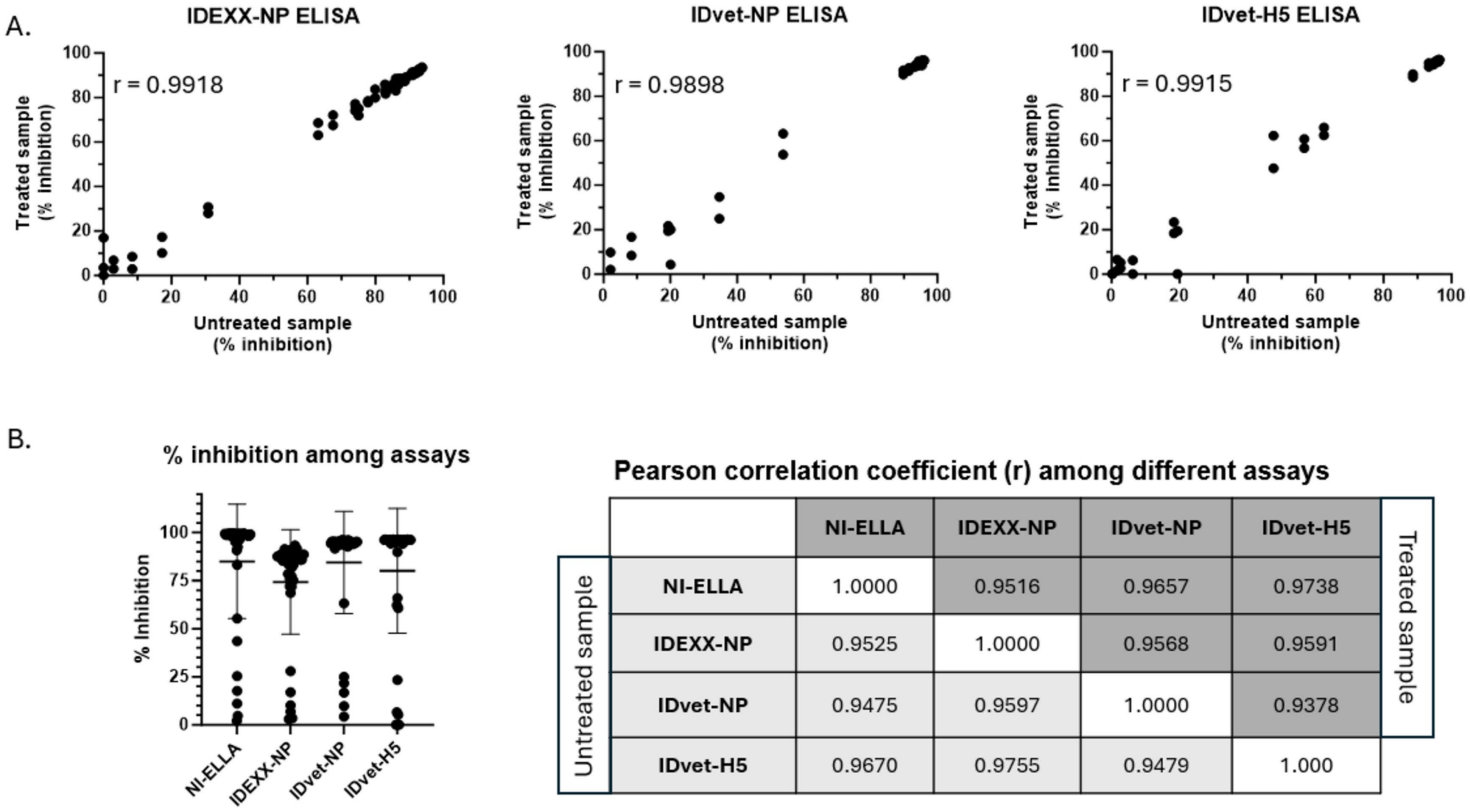
Comparison of untreated and treated milk samples using commercial ELISA kits **(A)** and correlation among NI-ELLA and ELISAs **(B)**. A. All samples were tested at a 1:5 dilution and percent inhibition values are shown. B. Percent inhibition values and Pearson correlation coefficients (two-tailed test) among the assays are shown.

Using the same 41 samples tested for endpoint titration of NI-ELLA, we evaluated the effect of sample treatment on commercial ELISAs commonly used for influenza A antibody detection. As shown in Figure 3, sample treatment did not alter results of any of the commercial ELISAs, and correlations between treated and untreated samples were high (*r* > 0.98; Figure 3A). Correlations among different ELISAs and NI-ELLA were also strong (*r* > 0.93; Figure 3B). These findings support the use of heat-treated infranatant samples for ELISA-based assays when sample quality is uncertain.

### 3.4 Comparison of NI-ELLA and IDEXX-NP ELISA

**Figure 4:**
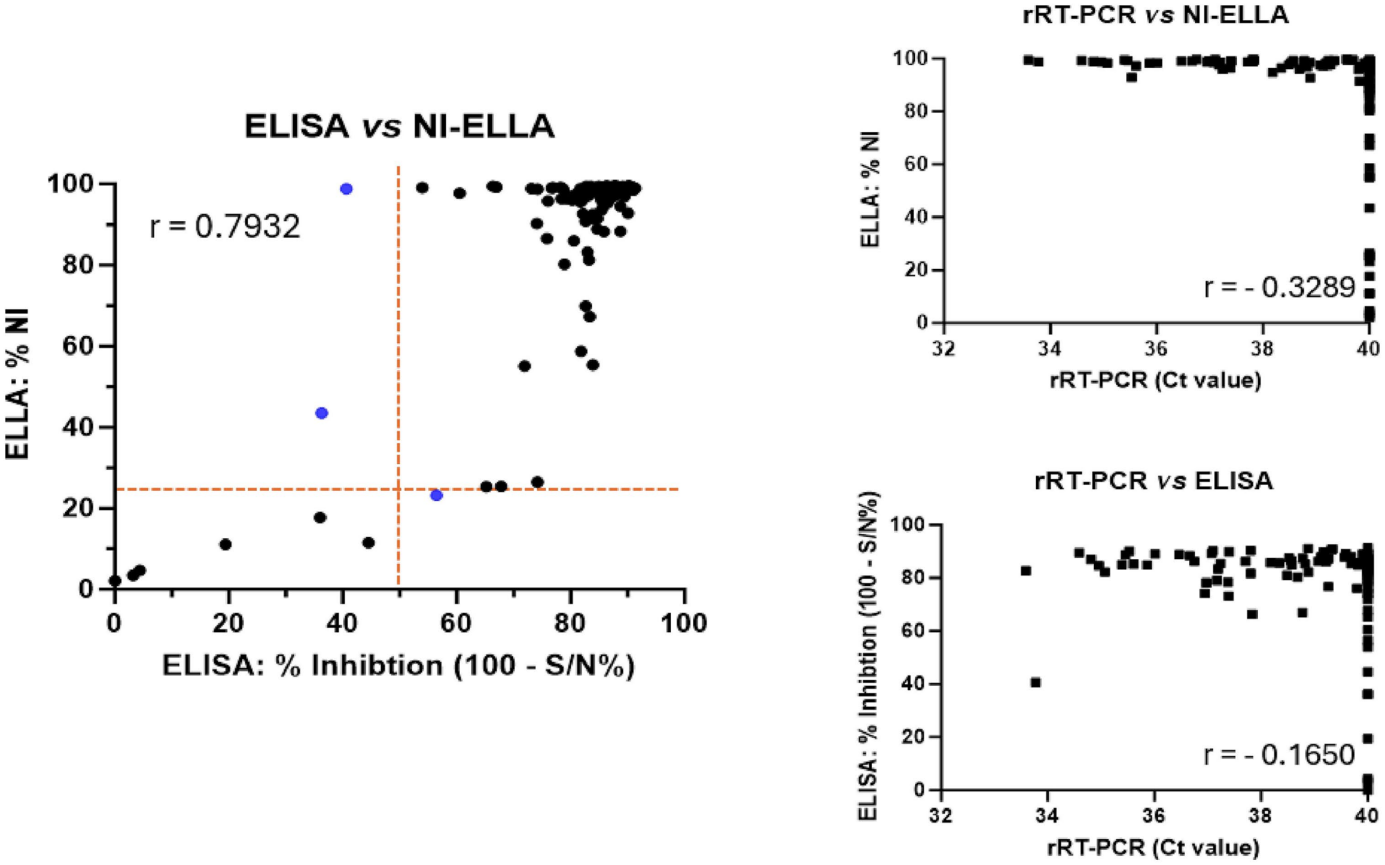
Comparison among NI-ELLA, IDEXX-NP ELISA, and rRT-PCR data. *Left panel:* Percent inhibition values obtained with NI-ELLA and ELISA are plotted. Untreated milk sample at a 1:20 dilution was used for NI-ELLA, and undiluted milk was used for ELISA. Blue dots represent three discrepant samples positive in only one test; black dots (*n* = 107) represent samples with concordant results. *Right panel*: Cycle threshold (Ct) values from rRT-PCR and percent inhibition values from NI-ELLA or ELISA are plotted. Ct values represents the number of amplification cycles required for viral RNA detection, and a value of 40 indicates no detectable viral RNA amplification during the 40 PCR cycles. Pearson correlation coefficients (*r*) (two-tailed test) are shown.

Milk samples collected from HPAI virus affected herds were tested by rRT-PCR and IDEXX NP-ELISA at NVSL using untreated, undiluted samples. We compared NP-ELISA results with NI-ELLA performed at a 1:20 dilution. The NP-ELISA positive cut-off is 50% inhibition per manufacturer’s direction, and the NI-ELLA cut-off was set at 25% NI reactivity based on the mean + 3 standard deviation (23.97%) of untreated antibody-negative samples tested (Figure 1). Based on these criteria, 107 of 110 samples (97.3%) agreed on test results between NP-ELISA and NI-ELLA, while 1 and 2 samples were negative by NI-ELLA and NP-ELISA, respectively, while other tests were positive (Figure 4, left panel). Pearson correlation was strong (*r* = 0.7932) despite the use of undiluted samples for NP-ELISA, which can generate high, non-differential percent inhibition values. Comparing endpoint titers between the assays may have provided better insight, but the endpoint titration of a large number of samples using commercial ELISA kits is cost-prohibitive.

We also compared NVSL rRT-PCR data by correlating cycle threshold (*Ct*) value obtained via rRT-PCR with the levels of antibody detected by NI-ELLA and ELISA. No meaningful correlation was observed between *Ct* values and antibody levels (Figure 4, right panel).

### 3.5 Comparison of antibody titers from NI-ELLA and VN test

**Figure 5:**
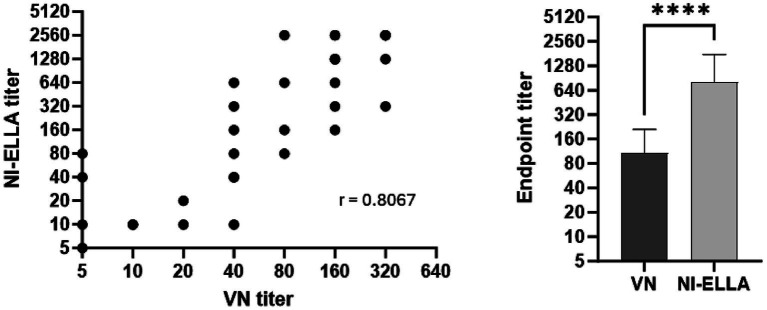
Comparison of virus neutralization (VN) antibody titers and NI-ELLA titers of selected milk samples (*n* = 41). The Pearson correlation coefficient (*r*) (two-tailed test) is shown in the left panel. The right panel shows the comparison of mean endpoint titers obtained with the VN test and NI-ELLA. For VN and NI-ELLA titers, any titer below 10 was assigned a value of 5, and any titer below 20 was assigned a value of 10, respectively. Paired *t*-test was conducted. For the comparison of titers between the assays, both the direct and log₂-transformed data were analyzed, and both yielded identical significance levels. ****p < 0.0001.

VN test is currently the reference method for measuring protective antibody levels in milk samples, and untreated samples are typically used for the assay (12, 19). The same 41 selected samples were evaluated by VN tests. However, because 4 of the milk samples showed bacterial contamination, the endpoint titration based on CPE of MDCK cells could not be evaluated for those samples. Thus, VN antibody titers of 37 samples were determined and compared with NI-ELLA titers (Figure 5). A strong positive correlation between NI-ELLA and VN titers was observed (*r* = 0.8067), and the NI-ELLA titers were significantly higher than the VN antibody titers, consistent with results from poultry studies comparing NI-ELLA with HI titers in serum samples (22, 24).

### 3.6 Application of NI-ELLA for bulk tank milk samples

**Figure 6:**
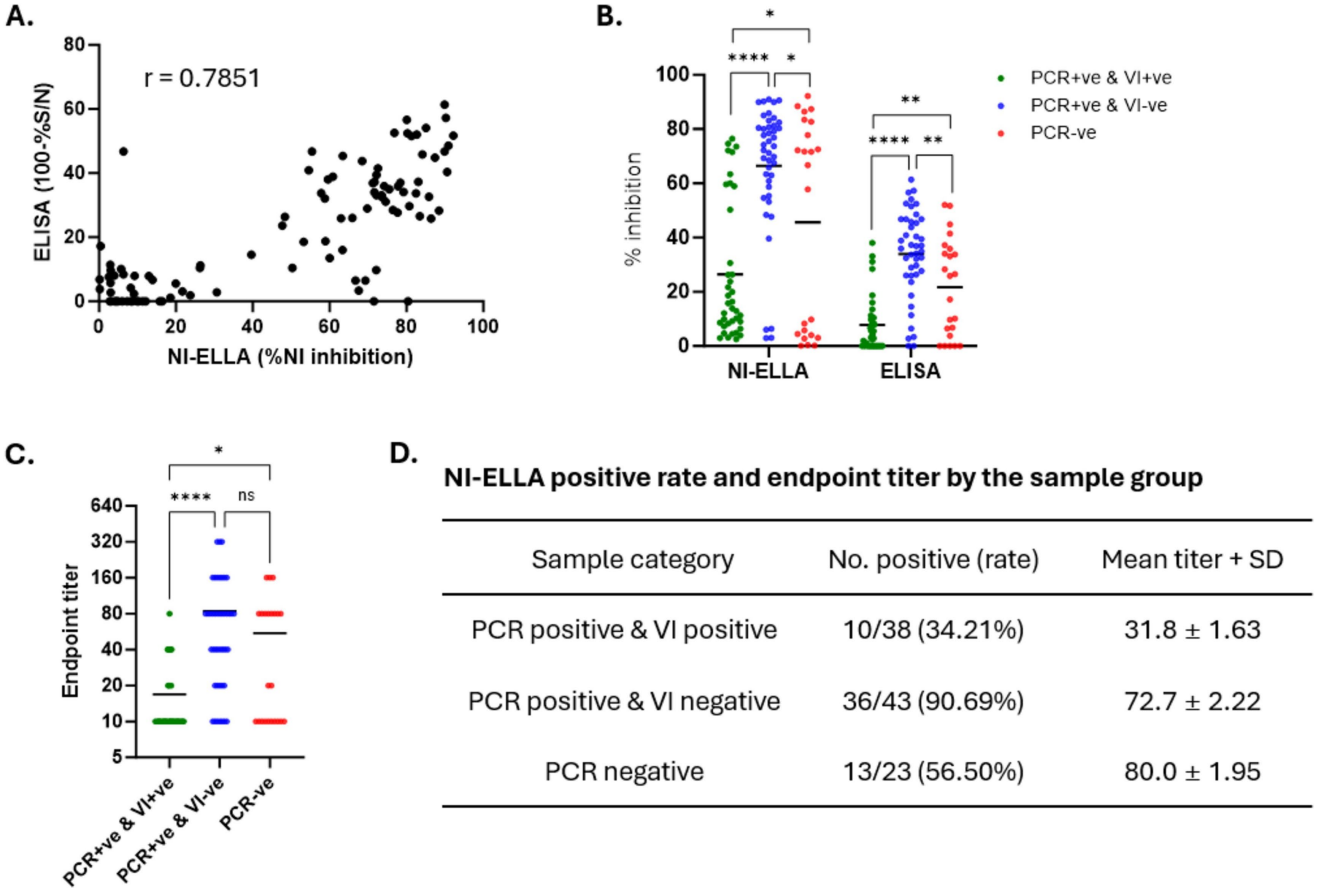
Comparison of NI-ELLA, ELISA, virus isolation and rRT-PCR results using bulk tank milk samples. Samples were categorized as rRT-PCR positive and virus isolation positive (PCR+ve and VI+ve), rRT-PCR positive and virus isolation negative (PCR+ve and VI−ve), and rRT-PCR negative (PCR−ve). **(A)**. Comparison of percent inhibition between NI-ELLA and ELISA, **(B)**. Comparison of mean percent inhibition of NI-ELLA and ELISA across sample categories, **(C)**. Comparison of mean NI-ELLA 50% endpoint titers among different sample categories, **(D)**. Comparison of NI-ELLA positive rates and mean titers (positive samples only) across sample categories. Ordinary ANOVA followed by Tukey’s post-hoc test was done for multiple comparison. For the comparison of titers among different sample categories, both the direct and log₂-transformed data were analyzed, and both yielded identical significance levels. ns, not significant; *p < 0.05; **p < 0.01; ***p < 0.001; ****p < 0.0001.

Individual milk samples tested in this study were all virus isolation negative samples, and correlating rRT-PCR data with serologic data did not yield meaningful insights (Figure 4). Therefore, we analyzed bulk tank milk samples previously collected for a different study (27). Due to variable sample quality and multiple freeze–thaw cycles, only centrifuged and heat-inactivated samples were tested, and both NI-ELLA and NP-ELISA were run at a 1:20 dilution. Because ELISAs were conducted at a higher dilution (1:20) of samples than manufacturer’s recommendation, we did not compare positive rate between the assays. Nonetheless, other than significantly higher percent inhibition values observed with NI-ELLA than NP-ELISA as expected, the two assays correlated well (*r* = 0.7851; Figure 6A), consistent with individual milk sample results (Figure 4).

Bulk tank milk samples were categorized as rRT-PCR positive and virus isolation positive (PCR+ve and VI+ve), rRT-PCR positive and virus isolation negative (PCR+ve and VI−ve), and rRT-PCR negative (PCR−ve) samples. Virus isolation-negative samples, including PCR−ve samples, clearly showed larger numbers of samples with significantly higher percent inhibition values than virus isolation-positive samples by both NI-ELLA and ELISA (Figure 6B). Endpoint titers determined by NI-ELLA were also significantly higher in virus isolation-negative samples, regardless of the rRT-PCR status, compared with virus isolation-positive samples (Figure 6C).

## 4 Discussion

The ongoing HPAI outbreak in US dairy cattle is unprecedented, and the impact of HPAI virus infection on the mammary gland, both in pathogenesis and as a potential source of viral spread, is extraordinary (9, 10). Therefore, milk samples are a valuable diagnostic fluid for detection of ongoing infection or prior exposure to HPAI virus (14, 15). Previous studies demonstrated the effectiveness of NP-based commercial ELISA for detecting antibodies in milk samples early in the HPAI H5N1 outbreak in dairy cattle (18), and more recently, H5-specific antibody detection by FMIA, which can be expanded to multiplex assay formats (17). Our study further demonstrates the applicability of milk samples for monitoring H5N1 HPAI infection and evaluation of the antibody response through detection of N1-specific antibody using NI-ELLA. As we have previously demonstrated in chicken studies (22, 24), this study also shows that NI-ELLA is a sensitive and cost-effective tool for quantification of antibody levels for evaluation of immune response that can support other functional assays such as the VN test.

It is highly desirable for the samples to be tested without treatment or processing, especially when frontline laboratories are testing large numbers of samples each day. Unlike serum samples, which typically require receptor-destroying enzyme (RDE) treatment to reduce nonspecific binding in different assays (29), milk samples have been tested both untreated (fresh, high quality samples), and as defatted samples through a combination of centrifugation and rennet treatment (17, 18). Our study supports previous findings that fresh milk samples may be tested without any treatment. In addition, our study shows that simple processing of samples (centrifugation) followed by heat treatment of infranatant can reduce the nonspecific reactivity in poor-quality samples, which were not kept frozen or have gone through multiple freezing and thawing cycles, without negatively affecting detection of N1-specific antibody by NI-ELLA and performance of commercial ELISAs.

Although this study did not assess NI-ELLA’s diagnostic specificity or sensitivity, the strong correlation between NI-ELLA and commercial NP-ELISA, which a previous study showed had high diagnostic specificity (18), provides evidence of concordance. Although not specifically addressed in this study, we previously demonstrated the superior sensitivity of the NI-ELLA compared to different types of commercial ELISAs for detecting H5N1 infection in chickens (21).

HI and VN tests are typically conducted as standard tests to measure antibody response in correlation with protective immunity from infection or vaccination (12, 19). We previously demonstrated a strong positive correlation between NI-ELLA and HI assay antibody titers, as well as higher sensitivity of NI-ELLA for evaluating HPAI and Newcastle disease vaccine-induced antibody responses (22, 24). This study further confirms the strong correlation between NI-ELLA and VN titers and shows great potential for NI-ELLA to serve as a sensitive, functional assay that can complement HI or VN tests for analyzing both serum and milk samples. By measuring endpoint titers of bulk tank milk samples by NI-ELLA, we were able to show a clear correlation between higher antibody titer and infectious virus negative samples (i.e., virus isolation negative), which most likely has to do with higher levels of neutralizing antibody in those samples. A higher antibody detection rate was also observed in virus isolation negative samples. Future studies are warranted to address other factors such as the possibility that high viral loads may interfere with antibody detection through the formation of antibody-virus complexes (30).

Although rRT-PCR serves as a highly specific and sensitive standard test for detecting active infection, the detection depends on the stage of HPAI virus infection. Thus, serological testing can be complementary to rRT-PCR in monitoring HPAI spread and epidemiological studies. The NP-based ELISA and N1 specific NI-ELLA are also complementary because they have different targets; NP-ELISA can detect any type A influenza virus infection and N1 NI-ELLA will specifically detect N1 subtype infection, which is expected to be H5N1 HPAI virus based on its current prevalence in US dairy cows. NI-ELLA can also be modified to identify any neuraminidase subtypes as needed. Furthermore, NP-ELISA and NI-ELLA can function as DIVA tests if vaccination is implemented in cattle (28).

Although this study provides valuable insights and demonstrates the potential application of NI-ELLA for HPAI monitoring in cattle, several limitations must be acknowledged. Full validation will require larger subsets of clinical samples, determination of an optimal cut-off (or threshold) using a receiver operating characteristic (ROC) curve, and comprehensive evaluation of diagnostic sensitivity and specificity. A recent study reported serological evidence of human and swine H1 and H3 influenza virus infections in different cattle breeds (31), and the implications of such infections in NI-ELLA performance should be further examined. This study also lacks longitudinal sampling, with limited information regarding timing relative to infection. Future studies are needed to characterize antibody dynamics and duration in milk samples over extended periods following infection in individual cows, for which NI-ELLA will serve as a valuable quantitative tool. Additionally, given the practical relevance of bulk-tank milk sampling, the effects of sample quality related to storage conditions and freeze–thaw cycles should be further investigated.

## Data Availability

The original contributions presented in the study are included in the article/supplementary material, further inquiries can be directed to the corresponding author.
